# Patient-Centred Management of Well-Controlled Haemophilia: Obtaining Opinions and Definitions Through a Delphi Consensus

**DOI:** 10.3390/jcm14103300

**Published:** 2025-05-09

**Authors:** Rubén Berrueco, Inmaculada Soto, José María Bastida, José Manuel Calvo Villas, Carmen de Cos, Saturnino Haya, Francisco Sierra García, José Mateo Arranz

**Affiliations:** 1 Hematología Pediátrica, Servicio de Hematología, Hospital Sant Joan de Déu, 08950 Barcelona, Spain; 2 Sección de Hemostasia y Trombosis, Servicio de Hematología y Hemoterapia, Hospital Universitario Central de Asturias (HUCA), 33011 Asturias, Spain; isotor60@gmail.com; 3 Departamento de Hematología, Complejo Asistencial Universitario de Salamanca (CAUSA), Instituto de Investigación Biomédica de Salamanca (IBSAL), Universidad de Salamanca (USAL), 37007 Salamanca, Spain; jmbastida@saludcastillayleon.es; 4 Unidad de Hematología y Hemoterapia, Hospital Universitario Miguel Servet, 50009 Zaragoza, Spain; jmcvillas@hotmail.com; 5 Unidad de Hematología y Hemoterapia, Hospital Universitario Puerta del Mar, 11009 Cádiz, Spain; mariac.cos@hotmail.es; 6 Unidad de Hemostasia y Trombosis, Servicio de Hematología, Hospital Universitari i Politècnic La Fe, 46026 Valencia, Spain; hagusat@gmail.com; 7 Servicio de Farmacia del Hospital de Torrecárdenas, 04009 Almería, Spain; francisco.sierra.sspa@juntadeandalucia.es; 8 Unidad de Hemostasia y Trombosis, Hospital de la Santa Creu i Sant Pau, 08025 Barcelona, Spain; jmateo@santpau.cat

**Keywords:** haemophilia, Delphi methodology, consensus, controlled patient, hemarthrosis, factor VIII deficiency, factor IX deficiency

## Abstract

**Background/Objectives**: For people with haemophilia, health-related quality of life mainly depends on the arthropathy caused by repeated joint bleeding. Prophylaxis is the standard of care in patients with severe bleeding phenotypes, but globally, none of the measures used to assess patients’ outcomes consider their desires and life expectations. We propose the concept of the “patient-centred management of well-controlled haemophilia” to define individual responses to prophylaxis. The aims of this work are (1) to achieve agreement about the definition of the “patient-centred management of well-controlled haemophilia” by a steering committee of experts, and (2) to share a series of statements that should define the “patient-centred management of well-controlled haemophilia” with other haemophilia clinicians looking for a consensus in this scenario. **Methods**: An eight-expert group was established to define the concept of the “patient-centred management of well-controlled haemophilia”. Seven major aspects were identified, and a final version of 42 statements was established and distributed to a 75-expert panel for consensus gathering using the Delphi methodology. **Results**: Forty-eight experts participated in the first round (participation rate: 64%); two sentences from domain 3 were split, leading to a total of 44 statements across the seven domains. Consensus was achieved in 92.85% of cases. Five items and three statements advanced to the second round. Eleven statements were reconsidered in the second round (response rate: 100%). The questionnaire presented high internal consistency. **Conclusions**: New treatments offer promising solutions for patients, but there is a paucity of models to measure global outcomes. Patient-centred medicine requires multidimensional assessment, and the “patient-centred management of well-controlled haemophilia” concept is intended to enable this.

## 1. Introduction

Haemophilia is an X-linked bleeding disorder caused by a defect in the synthesis and/or the function of factor VIII (FVIII; haemophilia A) or factor IX (FIX; haemophilia B), key components of the intrinsic pathway of blood coagulation [1]. People with haemophilia (PwH) are characterized by a bleeding tendency that usually correlates with low FVIII/FIX plasma levels. Thus, severe, moderate, and mild haemophilia are defined as <1%, 1–5%, and >5–40% of FVIII/FIX, respectively [2].

Bleeds can occur elsewhere, but health-related quality of life in PwH mainly depends on arthropathy derived from repetitive spontaneous and/or traumatic haemorrhages into joints and muscles, typically seen in severe and moderate patients [3]. To avoid this situation, long-term, regular prophylaxis, consisting of the regular administration of clotting factor concentrates to prevent haemorrhages while allowing PwH to lead an active life and achieve a quality of life (QoL) similar to people without haemophilia is recommended as the standard of care in PwH with severe bleeding phenotype [4]. Prophylaxis has demonstrated its utility in preventing joint bleeding and haemophilic chronic arthropathy [5]. However, despite prophylactic treatment, patients can still experience subclinical bleeds that can lead to joint damage [6]. Moreover, prophylaxis might also be associated with different downsides, such as the cost and the need for frequent injections.

To date, prophylaxis in haemophilia has consisted of the intravenous administration two or three times per week of standard half-life (SHL) plasma-derived or recombinant FVIII/FIX concentrates, a highly demanding schedule that has led to lack of adherence due to a variety of factors and barriers [7]. Recent advances in prophylaxis have changed the landscape of treatment for PwH, reducing patient burden, especially in high-income countries with access to treatment [8]. In fact, it should be taken into account that the treatment and care disparities described around the world have still led PwH to develop a higher rate of haemophilic arthropathy and disease burden [9]. Extended half-life (EHL) FVIII/FIX products have partially improved the frequency of factor infusions, especially for FIX, to every 5 to 14 days [10]. Emicizumab, a non-substitutive product that mimics FVIII administered subcutaneously every one, two, or four weeks, has been demonstrated to be useful for prophylaxis in PwHA with and without inhibitors of FVIII [11,12,13,14]. Moreover, upcoming and promising therapies focused on prolonging FVIII’s half-life and providing highly sustained factor activity [15,16,17], manipulating the balance between pro- and anticoagulant proteins, or introducing genetic material to PwH to potentially cure the disease may further improve patients’ outcomes [18,19].

The evaluation of these outcomes has been performed using different measures, like plasmatic factor levels, annual bleeding rate (ABR), annual joint bleeding rate (AJBR), joint health evaluation through physical examination and/or radiological studies, and patient-reported outcomes such us pain scales or health-related QoL (HRQL) questionnaires, among others [20]. While these tools provide valuable insights, each has inherent limitations, and a comprehensive assessment of PwH often requires a combination of multiple outcome measures [21]. Moreover, personalized medicine also includes information on these different expected outcomes for each patient and how to measure them. Available measures comprehensively evaluate PwH while encompassing different aspects, such their as clinical outcomes, social life, work, school, or psychological well-being, along with their personal desires and expectations. However, while numerous instruments assess specific psychosocial aspects such as anxiety, depression, treatment adherence, and compliance, there is no single tool that integrates all of these dimensions into a unified and individualized assessment [20,21,22]. Thus, a new concept such as that suggested by the authors of this work, “patient-centred management of well-controlled haemophilia”, should be established to better understand individual responses to prophylaxis.

The aims of this work were (1) to achieve agreement about the best definition of the “patient-centred management of well-controlled haemophilia” by a multidisciplinary steering committee of Spanish haemophilia experts, and (2) to share a series of statements that should define the “patient-centred management of well-controlled haemophilia” with other haemophilia clinicians in the country (expert panel) looking for a consensus in this scenario.

## 2. Materials and Methods

### 2.1. Project Flowchart

**Figure 1:**
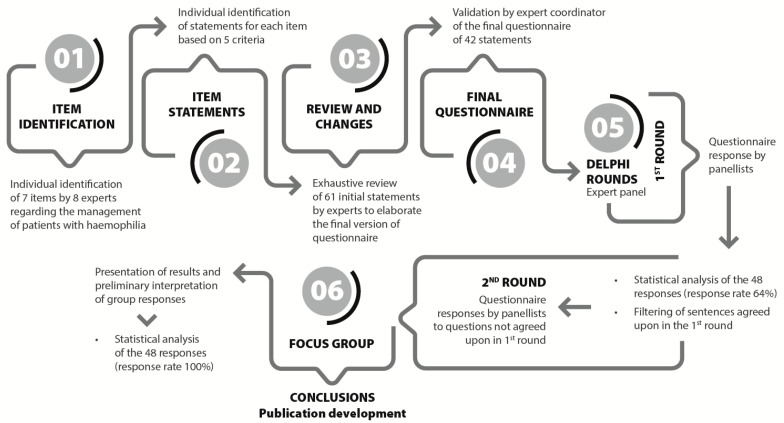
Project flowchart. Phases of the elaboration of the Delphi consensus questionnaire and rounds of response, which included a total of 42 statements related to the definition of “patient-centred management of well-controlled haemophilia”.

A focus group consisting of eight experts (six haematologist, one paediatric haematologist, and one pharmacist) was established based on their scientific knowledge and clinical expertise to define the concept of the “patient-centred management of well-controlled haemophilia” in haemophilia. Initially, a comprehensive literature review, with studies selected and shared by each participant, and several discussion meetings were conducted to identify seven major aspects related to the management of haemophilia patients apart from haemophilia severity, the treatment regimen, and the treatment used: disease burden, pain management, bleeding control, adherence, comorbidities, the patient’s perspective, and quality of life. Each of these domains was then thoroughly studied and analyzed in depth to draft concise and specific statements that would help to establish the concept of the “patient-centred management of well-controlled haemophilia”. This collaborative effort involved rigorous discussions, systematic reviews, and consensus-building exercises to ensure that the terminology was both comprehensive and precise. Each item was elaborated according to the following criteria: (i) short and specific sentences, (ii) clear and simple wording, (iii) potential controversy, and (iv) scientifically supported. This collaboration resulted in a questionnaire of 61 statements, which was later refined to a final version of 42 statements (Supplementary Table S1) (the project flowchart is shown in Figure 1).

An electronic questionnaire was distributed to an expert panel for consensus gathering using the Delphi methodology [23]. This study was based on the adequacy method of the RAND Healthcare Corporation and the University of California (Los Angeles, CA, USA). The methodology included two rounds of responses using a Likert Scale from 1 to 9. Scores of 1–3 indicated a consensus of disagreement, 4–6 that consensus was not reached, and 7–9 a consensus of agreement. A statistical analysis was carried out after the first round, filtering out the items on which consensus had been established. In the second round, the panellists were asked again to express their opinion only on the statements where consensus was not reached in the first round. Moreover, these statements could be revised and modified for clarity based on predefined criteria (Supplementary Table S1). After two rounds of responses, a descriptive analysis of all items was carried out followed by a new statistical analysis of the statements answered.

### 2.2. Expert Panel

Although there are no standard criteria for the selection of panellists, 75 experts were invited to participate in anonymous interactive voting based on their clinical experience and knowledge of haemophilia. Of them, 68 were specialists in haematology, 5 in hospital pharmacy, and 1 in paediatric haematology. Before answering the first round of statements, all selected experts were invited to respond to questions related to their daily clinical practice in haemophilia. Additionally, to investigate potential selection bias, other individual characteristics such as age, gender, and geographic distribution were identified.

### 2.3. Data Management

The answers to the questionnaire were received via an Excel database, ensuring that the data were captured accurately and stored in a structured manner for subsequent analysis.

Data management included an initial review of the responses, data cleaning to remove duplicates or incorrect entries, and preparation of the data for statistical analysis. Compatibility between the data formats in Excel and subsequent analysis tools was ensured, and any necessary transformations were documented to maintain data integrity.

For the statistical analysis, SPSS 28.0 analysis was conducted using ODBC provided within the software package (IBM Corp. Released 2021. IBM SPSS Statistics for Windows, Version 28.0, Armonk, NY, USA). This integration allowed for the direct importing of data from Excel to SPSS, ensuring that all variables and responses remained intact and ready for advanced analysis.

### 2.4. Statistical Analysis

To assess the internal consistency of the questionnaire, Cronbach’s alpha statistic (α of C) was used, ranging from 0 to 1. A score of 0 indicated no reliability, while 1 indicated maximum reliability. Scores above 0.7 were considered acceptable for our research purposes, those between 0.7 and 0.9 indicated high reliability, and those above 0.9 indicated very high reliability [24]. The intra-class correlation coefficient (ri) was calculated to assess inter-observer reliability: a score <0.40 is a poor correlation, 0.40–0.59 is moderate, 0.60–0.7499 is good, and >0.75 is excellent [25]. Both values were calculated for the entire questionnaire.

A descriptive analysis was performed for all items, including the mean ± SD and median values (25th–75th percentiles), the minimum and maximum values, and the statistical significance of the Kolmogorov–Smirnov goodness-of-fit test. Consensus on the statements was determined using agreement by terciles. If ≥2/3 of the responses fell in the tercile containing the median value, it was considered to indicate consensus, and discordance was indicated when ≥1/3 were outside the range containing the median. Consensus was further categorized based on the tercile containing the median value: the first tercile (values 1–3) indicates a consensus of disagreement with the statement; the second tercile (values 4–6) implies no consensus; and the third tercile (values 7–9) indicates a consensus of agreement with the statement.

Spearman’s correlation coefficient was calculated between the two rounds (by statement and domain). Qualitative agreement was assessed using the Kappa index for each statement, considering the groups of 1–3, 4–6, and 7–9. To evaluate the Spearman correlation scores, the criteria used were as follows: 0 to 0.25, low or null; 0.26–0.50, weak; 0.51–0.75, between moderate and strong; 0.76–1, between strong and very strong (perfect = 1) [26]. Meanwhile, the following scale of agreement was used to assess the Kappa index: <0.00, no agreement; 0.00–0.20, poor (or null); 0.21–0.40, weak; 0.41–0.60, moderate; 0.61–0.80, good; 0.81–1, very good [27].

The decision to proceed with subsequent rounds was based on the coefficient of variation (CV) of the total questionnaire scores in each round, together with the relative and absolute increase in the second round (CVsecond − CVfirst/CVfirst).

The protocol used was the same as that used in previous Delphi studies. The study adhered to the ethical guidelines outlined in the Declaration of Helsinki. All participants provided written informed consent.

## 3. Results

The study period for the Delphi consensus included two rounds of responses, conducted from 2 March 2023 to 11 September 2023 (Figure 1). Of the 75 Spanish experts invited to complete the questionnaire, 48 participated in the first round, resulting in a participation rate of 64%. After the initial round, two sentences from domain 3 were split, leading to a total of 44 revised statements across the 7 domains. Eleven statements were reconsidered in the second round, in which the questionnaire was completed by all the participants, yielding a 100% response rate. The mean age of the participants in the expert panel who voted in both rounds was 48.9 ± 11.8 years. The characteristics and profiles of the participating experts are shown in Table 1.

### 3.1. Internal Consistency of Questionnaire

The results showed that the questionnaire presented high internal consistency, nearing 0.9. However, there was variability in the consistency across domains, with some showing moderate values, due to a low number of statements in certain domains (Table 2).

### 3.2. Consensus by Terciles

Consensus was achieved in 39 items (92.85%) in the first round. Five items with values near or below 70% were considered borderline consensus and advanced to the second round, along with three statements where consensus was not achieved. One of these statements was divided into two separate items (S20).

The concordance among experts’ responses is detailed in Supplementary Table S2. One previously agreed-upon statement underwent further revision and was split into two (S21), resulting in a total of 11 statements for the second round. Consensus was reached for 10 out of these 11 statements (90.9%) (Table S3).

A high level of consensus was reached across all seven domains defined to characterize the patient-centred management of well-controlled haemophilia. The experts agreed that such a patients should experience minimal disease burden, good pain control, and individualized prophylaxis tailored to their clinical needs, preferences, and lifestyle. There was strong support for the early initiation of prophylaxis, the use of extended half-life factor concentrates, and the incorporation of validated tools to assess both bleeding control and pain management.

Adherence was recognized as essential, particularly in adolescents and young adults, with strategies like home delivery and simplified regimens seen as helpful. Experts also emphasized the relevance of including the patient’s perspective in treatment planning and the need to monitor comorbidities and quality of life regularly. While implementation may vary across settings, the consensus reflects a shared vision of comprehensive, personalized care in haemophilia. Full details of the consensus statements and their levels of agreement are provided in Supplementary Tables S1 and S3.

### 3.3. Inter-Round Correlation Analysis

In both the Spearman’s R and Kappa correlation values, a score equal to 1 correspond to items where no statements were present in the second round, as all statements reached sufficient consensus in the first round. The Spearman correlation values across domains ranged from moderate to very strong, suggesting a high quantitative correlation between the two rounds, both in the total scores and by domains. This is especially notable as it pertains only to statements without consensus. The Kappa index values across domains indicate qualitative agreement ranging from weak to good between the two rounds, with an overall score close to good for the entire questionnaire (Table 3). The Spearman correlation values by individual statement vary from weak to moderate/strong, suggesting that the changes in wording in the new statements generally resulted in relevant change in the answers.

When correlating the statements in the second round, statements 20 and 21 were split into two items (20a and 20b; 21a and 21b), and their values aligned with statements 20 and 21 from the first round, respectively. The Spearman correlation values per item ranged from low to moderate/strong, suggesting that the change in wording in the new statements generally led to relevant changes in the responses. The Kappa index values per statement suggested qualitative agreement ranging from null to weak, confirming relevant changes in the answers between the two rounds.

The CVs for both rounds were 0.17 ± 0.05 and 0.16 ± 0.05, respectively, with a relative delta of CV values <10% and of absolute values of <2%. These findings support the decision not to conduct a third round, as it would not result in a substantial change.

## 4. Discussion

This Delphi consensus project aimed to define the set of characteristics that determine whether haemophilia is well-controlled, based on the consensus of a group of Spanish experts in haemophilia. Rather than referring to intrinsic patient attributes, this concept reflects a clinical condition that results from meeting a series of agreed-upon criteria.

**Figure 2:**
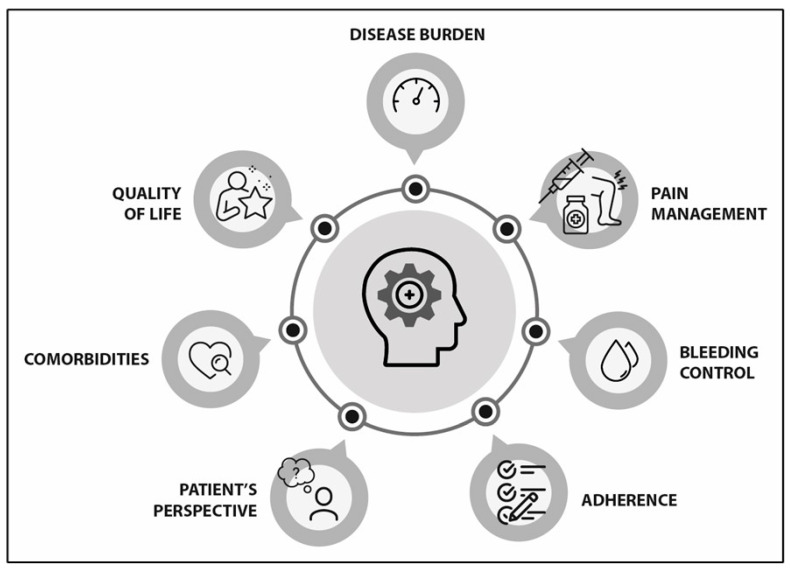
Main areas to be considered in patient-centred management of well-controlled haemophilia.

The landscape in haemophilia management is evolving very fast. New treatments achieve better therapeutic goals and patient outcomes. However, there is still room for improvement in the assessment of patient progress. Previous strategies have focused on developing a set of international standards to establish patient-relevant outcomes that should be evaluated in haemophilia [21]. However, the absence of a multidimensional tool capable of evaluating patient outcomes led a group of experts to assess in whom, when, and how haemophilia may be considered “well-controlled”. In the initial approach, the experts identified seven main domains to be considered when evaluating this new concept (Figure 2).

### 4.1. Disease Burden (See Supplementary Table S1, S1–S4)

The group of experts achieved a high level of consensus that well-controlled haemophilia should have a low disease burden. Haemophilia is a chronic condition characterized by a high dependency on specialized healthcare. Prophylactic treatment aims to normalize patients’ lives, prevent all types of bleeds, avoid complications, and improve QoL to levels similar to those of individuals without haemophilia while minimizing disease burden as much as possible [8,28]. To attain these objectives and address patient concerns, individualized treatment based on bleeding phenotype, joint health status, individual pharmacokinetics, physical activity, and patients’ preferences and lifestyle has been deemed crucial [4,7,25].

Additionally, the experts achieved consensus regarding the evolving needs of patients throughout life and emphasized the importance of patient and caregiver education for the social integration of paediatric patients. Active patient participation in shared decision-making with healthcare professionals has been shown to reduce disease burden. However, this approach requires a solid health literacy foundation and engagement in understanding available treatment options [29,30].

### 4.2. Pain Management (See Supplementary Table S1, S5–S13)

According to the group of experts, the “patient-centred management of well-controlled haemophilia” should include pain evaluation. Furthermore, PwH should either have no pain or be in good control of it. Apart from this, a high level of consensus was also reached on all the statements related to pain management in PwH. Nevertheless, a slight modification was needed for three of them before achieving expert consensus.

To optimize pain management in PwH, the panellists agreed that a regular evaluation should be conducted using questionnaires and pain assessment scales specifically validated for PwH [31,32].

Ideally, PwH should monitor any aspect related to their pain, such as type and severity, any changes to their treatment (including the use of opioids or other therapies) [31], the effectiveness of painkillers, and the impact of the pain on their quality of life. Moreover, healthcare professionals should also monitor the patients’ responses to pain treatment, any contraindications, and any adverse events related to pain therapy [31]. However, there is still a need to develop and validate standardized scales for recognizing and evaluating both acute and chronic pain in adults and children [33,34].

The current literature is scarce in terms of evidence-based pain management strategies tailored specifically for PwH. Current recommendations are predominantly based on guidelines intended for the general patient population without haemophilia and on the clinical experience of healthcare professionals treating PwH [35]. While specific recommendations for pain management in this population are required [31,32], the expert panel reached a high level of consensus on the preferred treatment in different clinical situations. These include the recommendation of the administration of oral paracetamol for acute hemarthrosis-related pain and the association of a weak opioid if an adequate response is not achieved during the next 4 h [4]. They also suggest using paracetamol as the first-line treatment for mild-to-moderate chronic pain related to arthropathy [4], and gradually introducing codeine or tramadol up to 3–4 times a day as second-line treatment if paracetamol is not effective enough [4,36]. Furthermore, COX-2-selective inhibitors are endorsed as both first- and second-line treatments for chronic pain in PwH [4,37].

The expert panel also recognized the pivotal role of physical medicine and rehabilitation in preventing and managing pain secondary to chronic arthropathy while enhancing and sustaining functional capacity [31,32,36]. However, the use of non-steroidal anti-inflammatory drugs (NSAIDs) for preventing the exacerbation of chronic arthropathy-related pain remains controversial, as consensus was not reached in the first round. Nevertheless, this discrepancy has been a topic of prior scientific debate. While the World Federation of Haemophilia (WFH) guidelines recommend using COX-2-selective inhibitors over NSAIDs for joint pain management in PwH [4,37], it has also been suggested that NSAIDs can be prescribed at the lowest effective dose for the management of short-term chronic articular pain associated with flare-ups [4,31,32,36,37]. In conclusion, the experts suggest that health professionals managing haemophilia would probably benefit from specialized guidelines and training in pain management tailored for this population.

### 4.3. Bleeding Control (See Supplementary Table S1, S14–S26)

The expert panel achieved a high level of consensus with the statement that well-controlled haemophilia should aim to have “balanced hemostasis” to prevent bleeds.

Consensus was also reached for the following key points: (1) Periodic prophylaxis started at an early age and administered at appropriate dosages to prevent bleeding episodes should be considered the gold standard for haemophilia management. This approach has been demonstrated to be superior to on-demand treatment to prevent joint disease and other associated complications [38,39,40]. (2) Prophylaxis should be adjusted on an individual basis, considering the patient’s bleeding tendency, joint health status, pharmacokinetics, preferences, and financial situation. (3) Web-based population pharmacokinetics tools are valuable for easily obtaining factor half-life and other important data using minimal blood samples [41]. (4) The variables with the most relevant impact on factor levels are the frequency of administrations and the half-life of the infused factor [42].

The individualization of prophylactic treatment would mean that if patients continue experiencing bleedings despite on-going treatment, the regimen should be gradually adjusted, increasing either the dose or the infusion frequency to prevent further haemorrhagic events. In this regard, the expert panel agreed that EHL FVIII/FIX has been demonstrated to improve outcomes compared to methods previously described with SHL [43,44,45,46]. However, following the initial consensus, the experts did not agree on the optimal strategy for optimizing prophylaxis. To address this divergence, the steering committee proposed two new statements covering both approaches. Thus, consensus was achieved.

Ultimately, the expert panel agreed that achieving zero bleeding episodes remains an unmet clinical need but a feasible goal with future therapeutic alternatives.

### 4.4. Adherence (See Supplementary Table S1, S27–S31)

Adherence to treatment was identified as another important area of evaluation in PwH. The World Health Organization (WHO) defines adherence as “the extent to which a person’s behavior—taking medication, following a diet, and/or executing lifestyle changes—corresponds with the agreed recommendations from a healthcare provider” [47].

Given that adherence includes compliance with healthcare professionals’ recommendations, there is an implicit need for a high level of collaboration between the patient and the multidisciplinary care team. The expert panel achieved a high level of consensus that adherence to treatment is more likely to be achieved when patients collaborate closely with their healthcare professional to devise an individualized therapeutic plan. The existing literature supports that suboptimal adherence is associated with poor outcomes in PwH, leading to a non-controlled disease (elevated ABR, impaired QoL, increased pain, and higher rates of work absenteeism) [48,49].

Consensus was also achieved regarding patient-specific characteristics that may compromise adherence, such as age. It is widely acknowledged that adolescents and young adults exhibit lower adherence rates [50]. Consequently, this demographic aspect should be considered to develop targeted intervention strategies to enhance adherence in specific subgroups of PwH. On the other hand, the expert panel also agreed that the implementation of certain strategies could be beneficial to improve adherence [51]. These include the use of EHL FVIII/FIX, the use of non-substitutive therapies, or the home delivery of treatment. Finally, despite the existence of adherence-specific questionnaires, their integration into routine standard clinical practice remains limited. Tools such as the VERITAS-Pro scale provide a validated method for the incorporation of such validated instruments into standard practice, representing an important field of research and clinical implementation to optimize adherence in PwH [52]. In addition, digital health technologies, such as mobile applications and electronic monitoring tools, have shown promise in tracking and improving adherence, offering a more dynamic and real-time approach to patient monitoring [53].

### 4.5. Patient Perspective (See Supplementary Table S1, S32–S34)

According to the expert panel, the definition of the “patient-centred management of well-controlled haemophilia” includes the patient perspective. It has been described that PwH perspectives change in response to improvements in the efficacy and safety profiles of new treatments [1,54]. According to the expert panel, a person with well-controlled haemophilia is defined as an empowered individual who is actively engaged in the management of their disease. Moreover, the expert panel concurred that the patient’s perception of disease control is influenced by the advent of innovative treatment that significantly enhances haemophilia management [51]. Consequently, the expert panel advocates for the standardization and validation of diverse tools designed to assess patient-reported outcomes (PROs). Such standardized tools would enable healthcare professionals to gain deeper insights into the patient’s perspective, thereby facilitating more patient-centred care [21,54].

### 4.6. Comorbidities (See Supplementary Table S1, S35–S38)

The generalization of prophylactic treatment as the standard of care has improved QoL and life expectancy in PwH. This longevity has been accompanied by an increase in different comorbidities, some related to haemophilia, such as arthropathy, human immunodeficiency virus (HIV) and hepatitis C virus (HCV) infections, and others related with ageing such as obesity, hypertension, cardiovascular diseases, metabolic diseases, and osteoporosis [55,56].

The expert panel achieved a high level of consensus in considering that well-controlled haemophilia should also exhibit appropriate management of potential comorbidities. To this end, three key statements were formulated regarding the most salient comorbidities in PwH: (1) Given that intracranial bleeding is a severe and a life-threatening complication in PwH, risk factors, such as hypertension, should be rigorously monitored in all patients, with particular attention paid to older patients (60–84 years old) with HIV infection receiving treatment with antiretroviral drugs [57]. (2) Atrial fibrillation risk increases with age, and its rate is higher in mild haemophilia patients [58]. Antithrombotic treatment versus other interventional alternatives should be evaluated individually considering the possible risks and benefits for each patient [59]. (3) Finally, thrombotic risk should also be evaluated on an individual basis; thus, pharmacological prophylaxis must be considered only in selected patients with high thrombotic risk. In fact, the WFH guidelines recommend adopting the same management strategies as those employed for non-haemophilic patients if these PwH are receiving adequate prophylactic therapy [4].

While the expert panel reached consensus on these three statements, it should be considered that as PwH get older, they become susceptible to a broader spectrum of age-related conditions, including cancer, diabetes mellitus, and dyslipidaemia. Haemophilia care providers should remain vigilant to all of these health challenges and actively participate in multicentre studies. Such collaborative efforts will enable healthcare professionals to formulate tailored management strategies for PwH, thereby optimizing their overall health outcomes.

### 4.7. Quality of Life (See Supplementary Table S1, S38–S42)

The expert panel also achieved consensus on the importance of the periodic evaluation of patient-reported QoL outcomes using validated questionnaires to determine whether haemophilia can be considered “well-controlled”. This aligns with the contemporary definition of prophylaxis in haemophilia, which aims to achieve a QoL similar to that of individuals without haemophilia [4]. Indeed, the overarching goal of healthcare professionals should be to enable PwH to achieve a “free-mind haemophilia status”, living a full life replete with the same expectations and aspirations as their peers [4,60,61].

Limperg PF et al. described the utility of using QoL questionnaires in PwH. Such a tool might facilitate open dialogue between healthcare professionals and patients regarding issues identified in the questionnaire responses that might otherwise remain unaddressed [62]. Despite the recognized value of QoL assessments, the expert panel agreed that QoL measurement remains non-standardized in clinical practice. Numerous questionnaires are available with varying levels of complexity, reliability, validity, and sensitivity, each providing diverse sets of information. Moreover, these tools need translations and cross-cultural adaptations that must be properly validated. Such variability complicates the implementation and comparability of these tools across different healthcare settings and countries [54,62]. It is important that healthcare professionals and patients collaborate to identify the most relevant outcomes for evaluation, determine the optimal frequency of assessments, and agree upon which temporal variations in results are indicative of effective disease control. A notable limitation highlighted by the consensus is the current inability to comprehensively assess mental health in PwH. These challenges underscore several unmet needs in the field that warrant further investigation and intervention.

### 4.8. Strengths and Limitations

This study has several limitations that should be taken into consideration, most of them intrinsic to the use of the Delphi method. The modified Delphi method is derived from a limited number of answers; therefore, the use of pre-established sentences and the inability to know the individual opinion of each panellist can potentially introduce bias [63]. An additional limitation of the Delphi method is that it relies on the number of respondents to the questionnaire. However, previous studies have indicated that the recommended number of participants should range from 7 to 30 participants, as a higher number does not increase the validity of the Delphi method but may increase its complexity and associated costs [64]. On the other hand, the selection of the panellists included mainly haematologists. This decision was based on the fact that, in Spain, they are mainly responsible for all haemophilia units.

Despite these limitations, the questionnaire showed a high degree of internal consistency, as well as a moderate/strong and very strong degree of correlation between the two rounds. Furthermore, the questionnaire showed a high degree of consensus on the main items defined by the initial working group, which supports the fact that these are necessary items to assess the definition of the “patient-centred management of well-controlled haemophilia”. These findings are of significant interest to haematologists, paediatricians, and other healthcare professionals involved in the specialized management of haemophilia patients. However, future work involving other stakeholders, such as healthcare professionals from other disciplines, payers, and caregivers/patients, will be necessary to validate and probably expand this definition.

### 4.9. Implications and Clinical Relevance

The results of this Delphi consensus provide a valuable framework for defining the “patient-centred management of well-controlled haemophilia”, which can have important implications in our clinical practice. This definition may facilitate more structured patient assessment, improve treatment individualization, and improve the decision-making processes. Nevertheless, we are aware that implementing all of these criteria within different healthcare systems may vary significantly depending on available local/national resources, different healthcare settings (public/private), and even institutional workloads. Unfortunately, this study did not evaluate feasibility, but the expert panel recognizes that the incorporation of all of these evaluation criteria can be challenging, especially if we aim to ensure their applicability across different hospitals and levels of care. However, we are certain that by addressing these challenges, we will optimize the management of PwH.

## 5. Conclusions

The concept of the “patient-centred management of well-controlled haemophilia” is a multidimensional approach that goes beyond widely known evaluation tools, integrating clinical, functional, and quality-of-life aspects. This Delphi study has helped to define the key criteria that might characterize well-controlled haemophilia, including bleeding control, pain management, treatment adherence, disease burden, patient perspective, comorbidities, and quality of life.

These findings can guide healthcare professionals to evaluate PwH and to help them to make more personalized treatment decisions. However, challenges remain in standardizing assessment tools, determining monitoring frequency, and validating these criteria in different clinical settings. The incorporation of digital health technologies and the integration of these concepts into routine practice could facilitate their implementation and contribute to more efficient, patient-centred care.

The next steps include the evaluation of the applicability of these criteria in different healthcare settings and their impact on clinical outcomes and the quality of life of patients with haemophilia.

## Figures and Tables

**Table 1:** Profile of responding experts.

	Rounds 1 and 2
Age (years) (mean ± SD)	48.9 ± 11.8
Years of experience (median (25th–75th percentiles))	12 (8–21)
Percentage of clinical practice time dedicated to haemophilia (median (25th–75th percentiles))	30 (20–50)
Percentage of patients receiving prophylaxis in moderate haemophilia (median (25th–75th percentiles))	37.5 (10–75)
Percentage of patients receiving prophylaxis in severe haemophilia (median (25th–75th percentiles))	100 (95–100)
Percentage of patients on SHL treatment (median (25th–75th percentiles))	15 (5–20)
Percentage of patients on EHL treatment (median (25th–75th percentiles))	79.5 (51–84)

SD: standard deviation; SHL: standard half-life; EHL: extended half-life.

**Table 2:** Consistency of the questionnaire.

	Round 1		Round 2	
	α of C (*p*)	ri (*p*)	α of C (*p*)	ri (*p*)
Disease burden (S1–S4)	0.593 (<0.001)	0.567 (<0.001)	0.593 (<0.001)	0.567 (<0.001)
Pain management (S5–S13)	0.582 (<0.001)	0.534 (<0.001)	0.617 (<0.001)	0.543 (<0.001)
Bleeding control (S14–S26)	0.708 (<0.001)	0.657 (<0.001)	0.573 (<0.001)	0.582 (<0.001)
Adherence (S27–S31)	0.665 (<0.001)	0.684 (<0.001)	0.669 (<0.001)	0.688 (<0.001)
Patient’s perspective (S32–S34)	0.728 (<0.001)	0.671 (<0.001)	0.743 (<0.001)	0.691 (<0.001)
Comorbidities (S35–S38)	0.492 (<0.001)	0.495 (<0.001)	0.492 (<0.001)	0.495 (<0.001)
Quality of life (S39–S42)	0.654 (<0.001)	0.621 (<0.001)	0.654 (<0.001)	0.621 (<0.001)
Total (42S and 44S in rounds 1 and 2, respectively)	0.882 (<0.001)	0.852 (<0.001)	0.868 (<0.001)	0.830 (<0.001)

The alpha of the C and ri values were calculated for the total questionnaire and for each of the domains.

**Table 3:** Correlation between rounds. Spearman’s rank values higher than 0.76 indicate strong/very strong correlation, and values of 0.51–0.75 indicate moderate to strong correlation.

	Spearman’s Rank Correlation	*p*	Kappa Index	*p*
Burden Disease (S1–S4)	1	-	1	-
Pain management (S5–S13)	0.771	<0.001	0.345	0.001
S9: The first-line pharmacological treatment of chronic arthropathic pain in patients with haemophilia is paracetamol.	0.514	<0.001	0.248	0.011
S10: In the second-line pharmacological treatment of chronic arthropathic pain in adult patients, the combination of paracetamol and a weak opioid is used.	0.587	<0.001	0.227	0.059
S12: Treatment of exacerbation of chronic arthropathic pain in patients with haemophilia may include oral non-steroidal anti-inflammatory drugs.	0.478	0.001	0.216	0.041
Bleeding (S14–S26)	0.771	<0.001	0.374	<0.001
S14: The controlled haemophilia patient should have balanced haemostasis.	0.156	0.288	0.030	0.279
S19: The most appropriate prophylaxis for optimal haemostatic protection is replacement therapy with deficient clotting factor concentrates for patients with haemophilia.	0.471	<0.001	0.205	0.049
S20a: One of the options for improving hemostatic protection with factor replacement therapy prophylaxis is to increase the administration frequency.	0.195	0.183	0.086	0.132
S20b: One of the options for improving hemostatic protection with prophylaxis by factor replacement therapy is to increase the dose and maintain the frequency of administrations.	−0.028	0.848	−0.059	0.514
S21a: One of the options to improve haemostatic protection with prophylaxis by factor replacement therapy is to use products with a longer half-life.	0.238	0.103	0.001	0.999
S21b: To improve haemostatic protection with factor replacement therapy prophylaxis, the frequency of administrations should be increased or products with a longer half-life should be used.	0.006	0.970	−0.072	0.438
Adherence (S27–S31)	0.930	<0.001	0.843	<0.001
S31: Home delivery of medication may improve adherence in patients with haemophilia	0.571	<0.001	0.603	<0.001
Patient Perspective (S32–S34)	0.891	<0.001	0.475	<0.001
S32: The haemophilia patient under control is one who is aware of the burden of the disease and the potential consequences	0.383	0.007	0.110	0.084
Comorbidities (S35–S38)	1	-	1	-
Quality of life (S39–S42)	1	-	1	-
Total (42S and 44S in rounds 1 and 2, respectively)	0.922	<0.001	0.781	<0.001

## Data Availability

The original contributions presented in this study are included in the article/Supplementary Materials. Further inquiries can be directed to the corresponding author(s).

## Supplementary Materials

The following supporting information can be downloaded at: https://www.mdpi.com/article/10.3390/jcm14103300/s1, Table S1: Wording of first and second rounds of statements; Table S2: Concordance of responses among group of experts; Table S3: Consensus and degree of agreement or disagreement.

## Abbreviations

The following abbreviations are used in this manuscript:

ABR	Annual bleeding rate
AJBR	Annual joint bleeding rate
CV	Coefficient of variation
EHL	Extended half-life
FVIII	Factor VIII
FIX	Factor IX
HCV	Hepatitis C virus
HIV	Human immunodeficiency virus
HRQL	Health-related QoL
NSAIDs	Non-steroidal anti-inflammatory drugs
PwH	Patients with haemophilia
PwHA	Patients with haemophilia A
QoL	Quality of life
SD	Standard deviation
SHL	Standard half-life
WFH	World Federation of Haemophilia
WHO	World Health Organization
