# Supplementary material for: Patient-Centred Management of Well-Controlled Haemophilia: Obtaining Opinions and Definitions Through a Delphi Consensus
Source: J Clin Med. 2025 May 9;14(10):3300. doi: 10.3390/jcm14103300 (PMC12111991; doi:10.3390/jcm14103300)
Supplement: Supplementary file 1 [file jcm-14-03300-s001.zip › jcm-3496092-supplementary.pdf]

Supplementary Appendix

Statements included in first and second round

A total of 42 sentences were developed, divided into 7 blocks in the first round, of which 9 were asked again in the second round: in 3 of them no agreement had been reached, 5 were at the limit of agreement (70%) and 1 had reached consensus. Of these 9, the statement was modified in 6 sentences (S9, S10, S14, S19, S31 and S32) and 2 of them were split into two (S20 and S21). The wording of all sentences in both the first and second round is shown below.

Table S1. Wording of First and second round statements.

N.º	Wording round 1
Disease burden	
S1	"W*ell-controlled* hemophilia" implies that a person with haemophilia suffers the lowest possible burden derived from the disease.
S2	The needs of the people with haemophilia change throughout their life course and should be adapted to their age and personal circumstances.
S3	Achieving optimal joint health and social integration in the child with haemophilia is mediated by education and the family's engagement with the disease.
S4	It is important to involve the patient in decision making related to his or her disease in order to offer the best available treatment for each situation.
Pain management	
S5	Healthcare professionals treating haemophilia should collect on regular basis the characteristics and severity of the pain of their patients, using validated assessment tools.
S6	It is important to differentiate between acute and chronic pain in people with haemophilia.
S7	The need for analgesic drugs should be assessed and their use by people with haemophilia should be recorded.
S8	The drug treatment of choice for acute haemarthrosis pain in the adult is oral paracetamol along with a weak opioid if the pain is severe (numerical pain scale (NRS) >7) or there is no therapeutic response in less than 4 hours.
S9	The first-line pharmacological treatment of chronic arthropathic pain in people with haemophilia is paracetamol.
S9 (2ºRonda)	*Paracetamol is used in the first-line pharmacological treatment of chronic arthropathic pain in people with haemophilia.*
S10	*The recommended second-line pharmacological treatment of chronic arthropathic pain in adult patients is the combination of paracetamol and a weak opioid.*
S10 (2ºRonda)	*In the second-line pharmacological treatment of chronic arthropathic pain in adult patients, the combination of paracetamol and a weak opioid is used.*
S11	*Management of chronic joint or muscle pain in people with haemophilia should be considered to include physical therapy and rehabilitation by healthcare professionals with expertise in haemophilia.*
S12	*Treatment of exacerbation of chronic arthropathic pain in people with haemophilia may include oral non-steroidal anti-inflammatory drugs.*
S12 (2º Ronda)	*Treatment of exacerbation of chronic arthropathic pain in people with haemophilia may include oral non-steroidal anti-inflammatory drugs.*
S13	The treatment of exacerbation of chronic exacerbated arthropathic pain in people with haemophilia may include COX-2 inhibitors as a first choice.
Bleeding control	
S14	*Controlled-haemophilia should have balanced haemostasis.*

S14 (2º Ronda)	*Controlled-haemophilia should have balanced haemostasis to avoid bleeding.*
S15	Controlled-haemophilia aims to avoid all type of bleeding episodes and to have pain control.
S16	Severe haemophilia phenotype is usually not well controlled if people with haemophilia they are treated with factor replacement therapy on demand.
S17	Early initiation of prophylaxis for severe haemophilia is the most important factor in preventing haemophilic arthropathy.
S18	Personalising haemophilia prophylaxis for a people with haemophilia involves considering together their clinical needs, personal preferences, pharmacokinetics and physical activity.
S19	The most appropriate prophylaxis for optimal haemostatic protection is replacement therapy with deficient clotting factor concentrates for people with haemophilia.
S19 (2nda Ronda)	For the highest protection from bleeds with prophylactic factor replacement therapy the alternatives are either to increase the frequency of administrations or to increase the half-life of factor.
S20	One of the options to optimize haemostatic protection with factor replacement therapy prophylaxis, requires increasing the frequency of administration.
S20a	One of the options for improving hemostatic protection with factor replacement therapy prophylaxis is to increase the frequency of administration.
S20b	One of the options for improving hemostatic protection with prophylaxis by factor replacement therapy is to increase the dose and maintain the frequency of administrations.
S21	One of the options to optimize the hemostatic protection with prophylaxis by factor replacement therapy is to increase the half-life of factor replacement therapy.
S21a	One of the options to improve haemostatic protection with prophylaxis by factor replacement therapy is to use products with a longer half-life.
S21b	*To improve haemostatic protection with factor replacement therapy prophylaxis, the frequency of administrations should be increased or products with a longer half-life should be used.*
S22	*The use of population pharmacokinetic models may allow for more precise adjustment of haemophilia prophylaxis.*
S23	Increasing factor target trough values is recommended in all patients to reduce the number of haemophilia haemarthroses, improve outcome and joint prognosis.
S24	Extended half-life factor concentrates in haemophilia patients reduce the number of infusions and costs vs the SHL.
S25	Extended half-life factor concentrates improve the results of standard half-life concentrates.
S26	Prophylaxis with extended half-life factor concentrates has been shown to improve patients' quality of life.
Adherence	
S27	Extended half-life factor concentrates allow for individualised prophylaxis regimens that improve adherence.
S28	Good adherence to treatment it is important for a better patient under control.
S29	In adolescents and young adults, the quality of adherence should be particularly monitored as they are more at risk of non-adherence.
S30	Reducing the number of times factor deficiency concentrate/non-replacement therapy is administered improves adherence.
S31	Home delivery of medication improves adherence in people with haemophilia.
S31 (2º Ronda)	Home delivery of medication may improve adherence in people with haemophilia.

Patient’s perspective	
S32	The well controlled-haemophilia implies that the person with haemophilia is aware of their disease and take responsibility over it.
S32	The well controlled-haemophilia implies that the person with is aware of the burden of the disease and the potential consequences.
S33	New treatments for haemophilia raise the patient's expectations for haemophilia control.
S34	Improved efficacy and safety of treatments increase the patient's perception of control.
S35	*The managed patient should have their non-haemophilia co-morbidities managed.*
S36	*In older people with haemophilia (60-84 years) and HIV treated with antiretroviral therapy (protease inhibitors), common risk factors involved in intracranial bleeding should be especially monitored.*
S37	*In people with haemophilia and atrial fibrillation, it is important to individually assess the risk-benefit of antithrombotic therapy or interventional alternatives to control atrial fibrillation (AF).*
S38	*Pharmacological prophylaxis of venous thromboembolism should be considered in selected patients at high thrombotic risk.*
S39	Quality of life questionnaires can indicate which people with haemophilia have a well-controlled disease.
S40	Measurement of quality-of-life questionnaires in haemophilia is not standardised in daily clinical practice.
S41	Collecting the results reported by people with haemophilia in the corresponding questionnaires helps to achieve the goal of a controlled disease.
S42	There is difficulty in assessing the mental health of people with haemophilia with currently available questionnaires.

Concordance by percentiles

To assess the concordance between the responses of group of experts for each sentence, the values of cases with data in the median range were calculated. A sentence (S) was considered to have a concordant response when <1/3 of the responses were outside that range, and discordance otherwise.

Table S2. Concordance of responses between group of experts.

	Round 1			Round 2		
	Median range	Participants in median range (%)	Type of concordance	Median range	Participants in median range (%)	Type of concordance
Disease burden						
S1	7-9	39(81.3)	Concordant	-	-	-
S2	7-9	48(100)	Concordant	-	-	-
S3	7-9	47(97.9)	Concordant	-	-	-
S4	7-9	48(100)	Concordant	-	-	-
Pain management						
S5	7-9	47(97.9)	Concordant	-	-	-
S6	7-9	48(100)	Concordant	-	-	-
S7	7-9	48(100)	Concordant	-	-	-
S8	7-9	39(81.3)	Concordant	-	-	-
S9	7-9	34(70.8)	Concordant (threshold)	7-9	38(79.2)	Concordant
S10	7-9	33(68.8)	Concordant (threshold)	7-9	36(75.0)	Concordant
S11	7-9	47(97.9)	Concordant	-	-	-
S12	7-9	29(60.4)	No Concordant	7-9	32(66.7)	Concordant (limit)
S13	7-9	44(91.7)	Concordant	-	-	-
Bleeding control						
S14	4-6	20(41.7)	No Concordant	7-9	47(97.9)	Concordant
S15	7-9	41(85.4)	Concordant	-	-	-
S16	7-9	38(79.2)	Concordant	-	-	-
S17	7-9	47(97.9)	Concordant	-	-	-
S18	7-9	47(97.9)	Concordant	-	-	-
S19	7-9	34(70.8)	Concordant (threshold)	7-9	32(66.7)	Concordant (limit)
S20	4-6	28(58.3)	No Concordant	-	-	-
S20a	-	-	-	7-9	40(83.3)	Concordant
S20b	-	-	-	7-9	31(64.6)	No Concordant (limit)
S21	7-9	37(77.1)	Concordant	-	-	-
S21a	-	-	-	7-9	48(100)	Concordant
S21b	-	-	-	7-9	46(95.8)	Concordant
S22	7-9	46(95.8)	Concordant	-	-	-
S23	7-9	43(89.6)	Concordant	-	-	-
S24	7-9	44(91.7)	Concordant	-	-	-
S25	7-9	46(95.8)	Concordant	-	-	-
S26	7-9	46(95.8)	Concordant	-	-	-
Adherence						
S27	7-9	43(89.6)	Concordant	-	-	-
S28	7-9	37(77.1)	Concordant	-	-	-
S29	7-9	48(100)	Concordant	-	-	-

S30	7-9	37(77.1)	Concordant	-	-	-
S31	7-9	34(70.8)	Concordant (threshold)	7-9	41(85.4)	Concordant
Patient's perspective						
S32	7-9	35(72.9)	Concordant (limit)	7-9	47(97.9)	Concordant
S33	7-9	46(95.8)	Concordant	-	-	-
S34	7-9	46(95.8)	Concordant	-	-	-
Comorbidities						
S35	7-9	44(91.7)	Concordant	-	-	-
S36	7-9	47(97.9)	Concordant	-	-	-
S37	7-9	48(100)	Concordant	-	-	-
S38	7-9	44(91.7)	Concordant	-	-	-
Quality of life						
S39	7-9	41(85.4)	Concordant	-	-	-
S40	7-9	41(85.4)	Concordant	-	-	-
S41	7-9	39(81.3)	Concordant	-	-	-
S42	7-9	44(91.7)	Concordant	-	-	-

In the first-round statements S20 and S21, the values of the corresponding second-round statement are shown split into two (S20a, S20b, S21a and S21b).

Consensus and agreement

Consensus if at least 2/3 are in the median range. A statement is in agreement when the median is in the groups 7-9 and disagreement when it occurs in the group of scores 1-3. Statements with answers 4-6 are considered indeterminate items.

Table S3. Consensus and degree of agreement or disagreement.

Statement	Round 1			Round 2		
	**Median range**	**Participants in median range (%)**	**Consensus/agreement**	**Median range**	**Participants in median range (%)**	**Consensus/ agreement**
Burden of Disease						
S1	7-9	39(81.3)	Consensus/agreement	-	-	-
S2	7-9	48(100)	Consensus/agreement	-	-	-
S3	7-9	47(97.9)	Consensus/agreement	-	-	-
S4	7-9	48(100)	Consensus/agreement	-	-	-
Pain management						
S5	7-9	47(97.9)	Consensus/agreement	-	-	-
S6	7-9	48(100)	Consensus/agreement	-	-	-
S7	7-9	48(100)	Consensus/agreement	-	-	-
S8	7-9	39(81.3)	Consensus/agreement	-	-	-
S9	7-9	34(70.8)	Consensus (threshold)/agreement	7-9	38(79.2)	Consensus/ agreement
S10	7-9	33(68.8)	Consensus (threshold)/agreement	7-9	36(75.0)	Consensus/ agreement
S11	7-9	47(97.9)	Consensus/agreement	-	-	
S12	7-9	29(60.4)	Non-consensus/agreement	7-9	32(66.7)	Consensus (threshold)/ agreement
S13	7-9	44(91.7)	Consensus/agreement	-	-	-
Bleeding						
S14	4-6	20(41.7)	Non-consensus/ undetermined	7-9	47(97.9)	Consensus/ agreement
S15	7-9	41(85.4)	Consensus/agreement	-	-	-
S16	7-9	38(79.2)	Consensus/agreement	-	-	-
S17	7-9	47(97.9)	Consensus/agreement	-	-	-
S18	7-9	47(97.9)	Consensus/agreement	-	-	-

S19	7-9	34(70.8)	Consensus (threshold)/ agreement	7-9	32(66.7)	Consensus (threshold)/ agreement
S20a	4-6	28(58.3)	Non-consensus/ undetermined	7-9	40(83.3)	Consensus/ agreement
S20b	4-6	28(58.3)	Non-consensus/ undetermined	7-9	31(64.6)	Non-consensus (threshold)/ agreement
S21a	7-9	37(77.1)	Consensus/agreement	7-9	48(100)	Consensus/ agreement
S21b	7-9	37(77.1)	Consensus/agreement	7-9	46(95.8)	Consensus/ agreement
S22	7-9	46(95.8)	Consensus/agreement	-	-	-
S23	7-9	43(89.6)	Consensus/agreement	-	-	-
S24	7-9	44(91.7)	Consensus/agreement	-	-	-
S25	7-9	46(95.8)	Consensus/agreement	-	-	-
S26	7-9	46(95.8)	Consensus/agreement	-	-	-
Adherence						
S27	7-9	43(89.6)	Consensus/agreement	-	-	-
S28	7-9	37(77.1)	Consensus/agreement	-	-	-
S29	7-9	48(100)	Consensus/agreement	-	-	-
S30	7-9	37(77.1)	Consensus/agreement	-	-	-
S31	7-9	34(70.8)	Consensus (threshold)/ agreement	7-9	41(85.4)	Consensus/ agreement
Patient Perspective						
S32	7-9	35(72.9)	Consensus (threshold)/ agreement	7-9	47(97.9)	Consensus/ agreement
S33	7-9	46(95.8)	Consensus/agreement	-	-	-
S34	7-9	46(95.8)	Consensus/agreement	-	-	-
Comorbidities						
S35	7-9	44(91.7)	Consensus/agreement	-	-	-
S36	7-9	47(97.9)	Consensus/agreement	-	-	-
S37	7-9	48(100)	Consensus/agreement	-	-	-
S38	7-9	44(91.7)	Consensus/agreement	-	-	-
Quality of life						
S39	7-9	41(85.4)	Consensus/agreement	-	-	-

S40	7-9	41(85.4)	Consensus/agreement	-	-	-
S41	7-9	39(81.3)	Consensus/agreement	-	-	-
S42	7-9	44(91.7)	Consensus/agreement	-	-	-

In statements divided in two in Round 2, both statements (a and b), the Round 1 column shows the same value of the corresponding statement of round 1.
